# A nucleosomal approach to inferring causal relationships of histone modifications

**DOI:** 10.1186/1471-2164-15-S1-S7

**Published:** 2014-01-24

**Authors:** Ngoc Tu Le, Tu Bao Ho, Bich Hai Ho, Dang Hung Tran

**Affiliations:** Japan Advanced Institute of Science and Technology, 1-1 Asahidai, Nomi, Ishikawa, 923-1292 Japan; Vietnam Academy of Science and Technology, 18 Hoang Quoc Viet, Hanoi, Vietnam; Hanoi National University of Education, 36 Xuan Thuy, Cau Giay, Hanoi, Vietnam

## Abstract

**Motivation:**

Histone proteins are subject to various posttranslational modifications (PTMs). Elucidating their functional relationships is crucial toward understanding many biological processes. Bayesian network (BN)-based approaches have shown the advantage of revealing causal relationships, rather than simple cooccurrences, of PTMs. Previous works employing BNs to infer causal relationships of PTMs require that all confounders should be included. This assumption, however, is unavoidably violated given the fact that several modifications are often regulated by a common but unobserved factor. An existing non-parametric method can be applied to tackle the problem but the complexity and inflexibility make it impractical.

**Results:**

We propose a novel BN-based method to infer causal relationships of histone modifications. First, from the evidence that nucleosome organization *in vivo* significantly affects the activities of PTM regulators working on chromatin substrate, hidden confounders of PTMs are selectively introduced by an information-theoretic criterion. Causal relationships are then inferred from a network model of both PTMs and the derived confounders. Application on human epigenomic data shows the advantage of the proposed method, in terms of computational performance and support from literature. Requiring less strict data assumptions also makes it more practical. Interestingly, analysis of the most significant relationships suggests that the proposed method can recover biologically relevant causal effects between histone modifications, which should be important for future investigation of histone crosstalk.

## Background

Genomes of higher organisms are organized into chromatin, a condensed structure of nucleosome units. Each of these units comprises a short piece of DNA wrapping around an octamer histone, containing two proteins of each type: H2A, H2B, H3, and H4 [1]. The histone protein is subject to various biochemical modifications, a.k.a. posttranslational modifications (PTMs), which have been shown to play crucial roles in many cellular processes, such as transcription and replication [2]. Defects of PTMs have also been implicated in determining cell fate and oncogenesis [3, 4]. The facts that PTMs may cause combinatorial effects on downstream events, and, by forming stable chromatin domains, properly pass modified states to the next generation [5, 6] suggest the existence of "histone codes" [7]. Therefore, revealing genome-wide PTM patterns and related functional implications would help increase our understanding of different DNA-mediated processes. For example, [8] discovered a common modification module of 17 modifications in human, suggesting their critical roles in gene regulation.

Advances in profiling techniques, such as ChIP-Chip and ChIP-Seq, have enabled the availability of genome-scale PTM data [8, 9], thus providing an unprecedented opportunity to decipher histone codes and their associated *cis*-regulatory elements. However, it also poses a great requirement for methods to understand such data. Many methods, ranging from clustering- to Hidden Markov Model (HMM)- to Bayesian network (BN)-based, have been developed to identify histone modifications patterns from ChIP-Chip and ChIP-Seq data [10–14]. Among them, BN-based approaches may help discover not only the cooccurrence but also the causal relationships of histone modifications [15]. This is especially important to understand histone crosstalk, a phenomenon that often occurs among different PTM events [16–18].

Bayesian network is a family of graphical models representing conditional independence of multiple variables [19]. First introduced to model gene regulatory networks (GRNs) from expression data [20], it has been widely used in reconstructing various biological networks, such as protein-protein interactions, protein signaling networks [21–23]. Likewise, there have been attempts to employ BNs to analyze histone modification data, in which compelled edges of the resulting models were considered causal relationships between PTMs [14, 24, 25]. Though useful, these works have a significant drawback: they require *causal sufficiency* assumption, i.e., all confounders of PTMs should be observed [26, 27]. This assumption, however, is unavoidably violated given the fact that some modifications can be regulated by enzymatic activity of a common but unobserved modifier [2].

**Figure 1:**
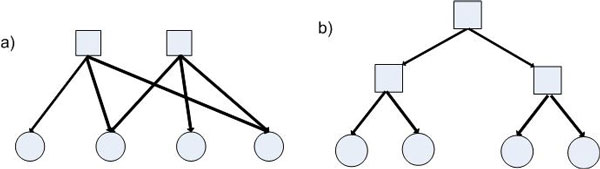
**Overlapping (a) and hierarchical (b) topologies**. The circles represent observed variables, the squares represent hidden ones.

Therefore, in order to reveal causal relationships of PTMs the existence of hidden confounders should be taken into account. Basically, there are two choices for network topology containing hidden confounders: overlapping and hierarchical [28]. In the overlapping (Figure 1a), each hidden variable is a parent of several observed variables, and several hidden variables can share a common observed variable as their child. In the hierarchical (Figure 1b), hidden variables form a tree structure, in which each of them is a parent of several other variables (either observed or hidden) and serves to capture the dependencies among its children and between its children and other nodes in the network. Biological evidences have showed that some modifications can be regulated by a common regulator and vice versa [2, 29]. Overlapping topology, therefore, is more suitable to describe the relationships between PTMs and their hidden regulators. Thus, the problem of learning network models representing causal relationships of PTMs can be formulated as learning two adjacency matrices, one representing the relationships among observed variables (PTMs), denoted as *X*, and the other representing the relationships between PTMs and their hidden causes, denoted as *Z*, as proposed by [30]. However, their non-parametric approach to learning the models requires strict data assumptions and employs a time-consuming procedure to infer *Z*. These drawbacks make it inflexible and inefficient in practice.

In this work, we propose a novel BN-based method to infer causal relationships of PTMs that accounts for the existence of hidden confounders. First, an information-theoretic criterion is proposed to selectively introduce a *pairwise hidden confounder* (PHC) for each pair of PTMs. *General hidden confounders* (GHCs) are then derived from PHCs. The idea of deriving GHCs from PHCs has been presented in [31] to learn two-layer BNs with hidden variables. Differently, we based our approach on the evidence that chromatin *in vivo* imposes regulatory effects on the activities of PTM regulators. Thus, the criterion is proposed exploiting information about chromatin structure, i.e., nucleosome positioning. Matrix *X* is separately learned by a BN structure learning method. Compelled edges, i.e., causal relationships, are then derived from a network model of both PTMs and GHCs. Application on human epigenomic data of 38 histone modifications and histone variant H2A.Z, shows that the proposed method outperformed the non-parametric (*Np*) and the traditional one, which does not account for hidden confounders (*noHidden*), in terms of computational performance and literature support. Moreover, analysis of the most significant relationships shows that the proposed method can recover biologically relevant causal effects between histone modifications, such as *H*3*K*27*Me*3 *→ H*3*K*9*Me*3, *H*3*K*4*Me*3 *→ H*2*AK*5*Ac*, *H*4*K*8*Ac → H*2*AZ*. This is important for future investigation of histone crosstalk.

## Methods

### Information theory

Mutual information (MI) has been increasingly used in reverse engineering, especially to reconstruct GRNs [32–35]. It is a more general measure compared to correlation in estimating the dependency between two variables. Given two random variables, *x* and *y*, MI is computed by:1

where *p*(*x*, *y*), *p*(*x*), and *p*(*y*) are joint density function and marginal density functions of *x* and *y*, respectively.

Likewise, conditional mutual information (CMI) is introduced to measure conditional dependency between two variables given the other(s). CMI of *x* and *y* given **z** (uni- or multi-variate) is computed by:2

If *x*, *y*, **z** are discrete variables, the integrals are replaced by the sum over all of their values. It is, however, difficult to compute the integrals given the limited number of samples in general cases. Thus, in practice, probability density functions are often approximated by density estimation methods. Given N samples of a variable **x**, density function can be approximated by:3

where *δ*(.) is the Parzen window function, **x**_*i*_ is the *i*th sample, and *h* is the window width. In our work, *δ*(.) was chosen as Gaussian function:4

where **z** = **x***−***x**_*i*_, *d* is the dimension of **x**, and Σ is the covariance matrix of **z**. When *d* = 1, equation (3) returns the estimated marginal density. When *d* = 2, it can be used to estimate the joint density function of bivariate variable (*x*, *y*). In our work, MI and CMI values were computed using a software package provided by [36].

### Bayesian networks

#### Definition

A Bayesian network is a directed graph representing conditional independence of multiple variables by a set of conditional probability distributions [19, 37]. Joint probability distribution of a variable set **x** encoded by the model can be factorized as:5

in which *p* (*x*_*i*_*|***Pa**_*i*_) corresponds to the local probability distribution of variable *x*_*i*_, and **Pa**_*i*_ are *x*_*i*_'s parents.

#### D-separation property

**Figure 2:**
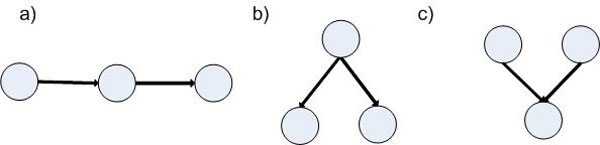
**Fundamental causal structures in BN models: serial (a), diverging (b), and converging (c)**.

In a BN, there are three fundamental local structures, namely *serial*, *diverging*, and *converging* connections (Figure 2). These structures are associated with a set of rules, which is independent of any particular calculus for certainty, to assess how a change of certainty in one variable may change the certainty for other variables in the networks. These rules form d-separation property of a BN. If two variables are d-separated, change in the certainty of one variable has no impact on the other. Two variables are called d-connected if they are not d-separated [19]. Thus, d-separation property can be used as a general assessment of the dependencies among nodes of a BN.

#### BN structure learning

BN structure can be learned by score-based methods, aiming to identify the structure(s) that "best" describe the data. In this work, BDe score [37, 38] with uniform prior was used to measure the fitness of a candidate network. Because it is infeasible to search though all possible structures [39], greedy hill-climbing search combined with simulated annealing algorithm to avoid local maxima was employed.

### Criterion for introducing PHCs

**Figure 3:**
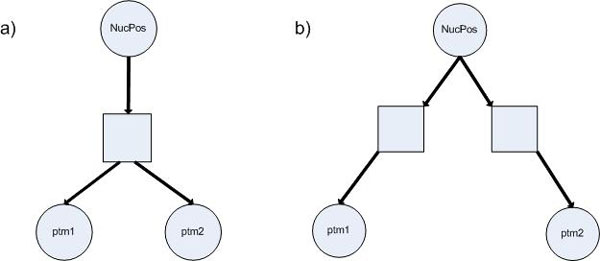
**Causal structures when two PTMs share a hidden confounder (regulator) (a) or not (b)**.

It has been widely shown that the binding of chromatin modifiers, and the large multiprotein complexes in which they reside, to chromatin is greatly affected by chromatin structure, i.e., nucleosome organization [7, 40–44]. From this observation, the relationships among two PTMs, their hidden regulator(s), and NucPos can be described by two local causal structures, illustrated in Figure 3. The following results can be easily proved based on d-separation properties:

**Proposition 1***Consider two PTMs*, *if each has its own (hidden) regulator*, *they will be d-separated given evidence on nucleosome positioning*.

**Proposition 2***Consider two PTMs*, *if they share a hidden regulator (in other words*, *a confounder)*, *their d-separation property does not change upon the availability of nucleosome positioning evidence*.

The results suggest that, given evidence on NucPos, the dependency level between two PTMs would not change if they share a hidden confounder, and would change (becoming "less" dependent) if each has its own (hidden) regulator. Using MI and CMI as the measures of dependency levels between two PTMs, we derive the following criterion for introducing a PHC for a pair of modifications, *ptm*1 and *ptm*2:

Define *Mutual Information Gain (MIG)* of two PTMs as:6

Then, a PHC is introduced if the following conditions are satisfied:7

where *α*, *β >*0 are significant thresholds. These criteria will be used to derive PHCs for all pairs of PTMs.

### Derivation of GHCs

From a set of PHCs derived in previous step, we define *hidden confounder graph*, an undirected graph whose nodes correspond to PTMs and edges to PHCs, implying that two nodes are connected if they share a PHC. Maximal clique algorithm is then applied on this graph, resulting in a set of maximal cliques, each corresponding to a GHC.

### Causal relationship inference

To derive causal relationships of PTMs, we first combine BN received from structure learning step with GHCs, forming a network of PTMs and their hidden confounders. The edges among PTMs that share a GHC are then removed. Finally, the algorithm for finding compelled edges [26] is applied to the resulting structure, producing a set of compelled edges representing causal relationships of PTMs.

### Data

**Chromatin modification**. CD4+ T cell data containing 20 methylations, 18 acetylations, and histone variant H2A.Z were retrieved from [9] and [8].

**Nucleosome positioning** data of resting CD4+ T cell was obtained from [45].

**Gene set**. UCSC Known Genes were retrieved from UCSC Genome Browser [46]. After removing genes with duplicated or without U133P2 probe IDs, 12456 genes were kept for analysis.

## Results

### Derivation of hidden confounders

**Figure 4:**
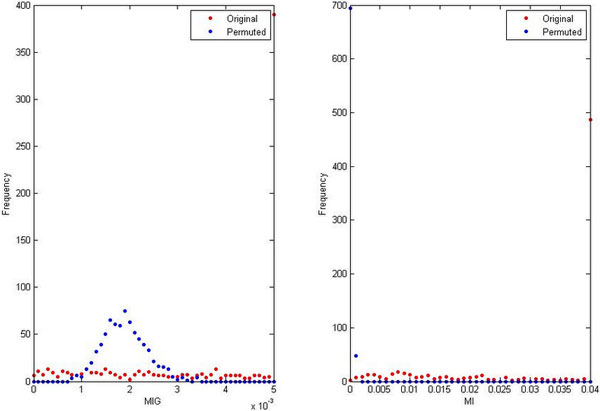
**Distributions of original and average permuted MIG values**.

Tag count profiles of 38 PTMs and histone variant H2A.Z, taken at the promoters (*TSS ±* 1*kb*) of 12456 selected genes, were first discretized into 3-category values. Tag count profiles of NucPos were transformed into logarithm scale. Then, all were used to compute *MI* and *MIG* values for all pairs of modifications. In Figure 4, the distributions of these values are illustrated in red. Permutation method [47] was employed to evaluate the significance of these distributions. By which, PTM profiles were permuted 1000 times and the distributions of the new *MI* and *MIG* values for all pair of PTMs were computed for each permutation. The averages of 1000 permuted *MI* and *MIG* distributions are illustrated in blue (Figure 4). The result showed that when *MIG ≤* 0.0007 and *MI ≥* 0.002, permutation was unable to create any association with the original *MIG* and *MI* distributions. The significant thresholds *α* and *β* were thus assigned to 0.0007 and 0.002, respectively. This resulted in a hidden confounder graph of 39 nodes and 63 edges. 50 maximal cliques were derived from this graph, corresponding to the same number of GHCs. The list of GHCs and their belonging modifications is given in supplementary information (http://www.jaist.ac.jp/~s1060011/SI.zip). Although it is hard to show that all GHCs are biologically relevant, we did find supporting evidences for some, whose child nodes are well-characterized modifications. For example, CBP is known to have enzymatic activity on both lysines 14 and 27 of histone H3 [2, 48], thus may play the role of confounder for H3K14Ac and H3K27Ac. The same observations were also reported for histone acetyltransferase GCN5, which may be the confounder of H3K14Ac and H3K36Ac [2, 49], or of H3K4Ac and H3K14Ac [2, 50]. Also, JMJD2C/GASC1 or JMJD2A/JHDM3A may be confounder of H3K9 and H3K36 methylation, though histone methyltransferases often target to specific residues [2].

### Inference of PTM causal relationships

#### General scheme

BN structures were learned by Banjo (http://www.cs.duke.edu/~amink/software/banjo/), limited to 1, 300, 000 iterations because no significant improvement was achieved in further iteration (data not shown). The resulted structures were combined with 50 GHCs derived in previous step to produce a set of causal relationships. Significance scores were evaluated by bootstrapping method [20]. By which, original data was randomly bootstrapped *N* times, generating *N* bootstrapped datasets, and a set of causal relationships was derived for each. Significance score of each relationship was defined as the frequency of its appearance in *N* bootstrapped sets. In our experiment, *N* was set to 100.

For comparison, the implementation of *Np* by [30] was run on the same data. Because it only works with binary variables, the data were discretized into binary values by three schemes, using 70 (Scheme 1), 80 (Scheme 2), and 90 (Scheme 3) percentiles as thresholds. After receiving hidden confounders, the above procedure was employed to generate three sets of causal relationships, corresponding to each scheme.

### Comparison

#### Performance

Table 1 presents the running time and number of hidden confounders derived by the two methods when running on a server machine (Intel Xeon X5570 2.93GHz (4 CPUs), 6GB RAM, Windows Server 2008 OS). It shows that, our method (denoted as *hidden*) worked faster than *Np* (converged after *≈* 200 iterations, data not shown) no matter what discretization scheme was employed. Moreover, to compute MIs and MIGs, it does not require any additional assumption on input data, thus more flexible and practical.

**Table 1:** Performances of Np and our method.

	***Np*** (200 iterations)			***hidden***
	**Scheme1**	**Scheme2**	**Scheme3**	
Running time (sec.)	5.0e+02	3.8e+02	2.8e+02	8.41
#Confounders	22	30	17	50

#Confounders is the number of hidden confounders.

#### Literature-based comparison

Because it does not exist a list of confirmed causal relationships that could be used as a "gold standard", we resorted to literature to compare the results given by different methods. Biomedical literature represents almost all of our existing knowledge about biological entities and their relationships. For the analysis presented here, we employed a simple but effective way to derive potential associations between PTMs from literature, the cooccurrence approach, which was previously applied for GRN reconstruction [51–53]. Simply, if two PTMs appear in an article abstract indexed in PubMed, we assume an association between them. However, in addition to the associations extracted based on direct cooccurrence, we also assume an association between two PTMs if they share some directly associated biomedical concepts. This assumption is based on the fact that PTMs often functionally interact with each other through intermediary proteins [2, 54]. To extract these indirect "associations" we employed FACTA+ [55], a state-of-the-art biomedical text mining system which supports both directly and indirectly related (pivot and target, respectively, so called in FACTA+) biomedical term search. Thus, two kinds of literature-based PTM associations were derived with the association weight defined as following. Regarding cooccurrence-based association, we took the weight definition from [52]:8

in which *freq*(*ptm*1, *ptm*2) is the frequency that both PTM terms appear together in PubMed abstracts, and *freq*(*ptm*_*i*_) is the frequency of each individually.

Regarding indirect association based on shared pivot concepts, i.e., proteins/genes in this case:9

in which *N* is the number of the most significant shared concepts between two PTMs, *sig*_1*i*_and *sig*_2*i*_are the significant levels, assigned as point-wise mutual information values, of the associations between the *ith* shared concept and the two PTMs. All of these were retrieved through FACTA+ search with the list of the search terms given in supplementary information.

**Figure 5:**
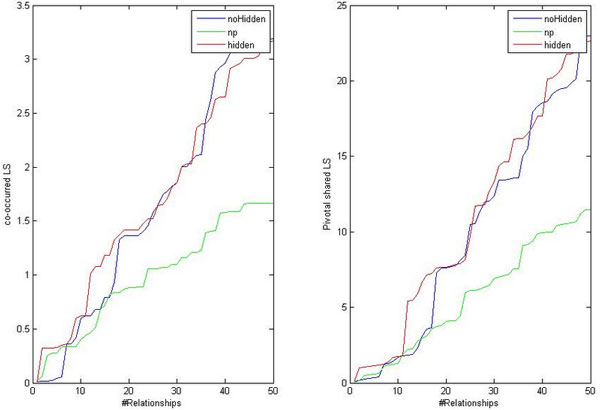
**Literature supports for the top 50 significant relationships given by our method (red),** ***Np*** **(green) (scheme 3), and** ***noHidden*** **(blue)**.

We define a measure, named *literature support*, for comparison purpose. It is the sum of literature-derived weights of *N* most significant associations (edges) of a resulting model *M*:10

where *w*(*e*_*i*_) is the literature-derived weight of the edge *e*_*i*_ (*i* = 1 *· · ·N*). Figure 5 illustrates literature supports for the top 50 significant relationships given by three methods. It shows that, in case of both direct (left figure) and indirect (right figure) associations, the most significant relationships given by our method have comparable literature support to the ones given by *noHidden*, and both are better than the result given by *Np*.

An alternative way for comparison is to assess the significance scores of PTM pairs previously reported as highly correlated [52]. [11] developed a biclustering method to search for combinatorial patterns of PTMs on the same data. From the resulting bilusters, they found three most frequently cooccurred PTM pairs: *(H*3*K*27*Ac*, *H*3*K*4*Me*3*)*, *(H*2*AZ*, *H*2*BK*120*Ac)*, and *(H*3*K*9*Ac*, *H*3*K*36*Ac)*. Also, we selected 10 most correlated PTM pairs (*r ≥* 0.7) reported by [8] in their pairwise correlation analysis on the data. Comparison on these two sets of highly correlated PTM pairs shows that the confidence scores assigned by our method are significantly higher than or at least equal to the ones assigned by the other two methods (Tables 2, 3, and supplementary information). This means, taking into account the existence of hidden confounders significantly increases our ability to recover highly correlated pairs of histone modifications.

**Table 2:** Comparison on the significance scores of three highly correlated PTM pairs reported in [11].

PTM pairs	***hidden***	***noHidden***	***p − value***
H3K27Ac-H3K4Me3	0.866	0.724	2.1e-10
H2AZ-H2BK120Ac	0.002	0.002	*Nd*
H3K9Ac-H3K36Ac	0.195	0.155	*Nd*

nd means no difference.

**Table 3:** Comparison on the significance scores of 10 most correlated PTM pairs reported in [8].

PTM pairs	***hidden***	***noHidden***	***p − value***
H2BK5ac-H3K27ac	0.677	0.481	6.26e-10
H2BK120ac-H2BK5ac	0.594	0.301	6.11e-10
H2BK120ac-H4K91ac	0.843	0.336	1.81e-15
H2BK5ac-H3K9ac	0.524	0.416	2.36e-08
H3K79me2-H3K79me3	0.794	0.793	*Nd*
H2BK120ac-H3K27ac	0.623	0.207	3.24e-13
H2BK120ac-H3K18ac	0.61	0.196	1.55e-14
H3K18ac-H3K27ac	0.453	0.19	1.28e-09
H2BK5ac-H3K18ac	0.047	0.004	4.31e-08
H2BK5ac-H4K91ac	0.294	0.283	*Nd*

nd means no difference.

### Analysis and discussions

**Figure 6:**
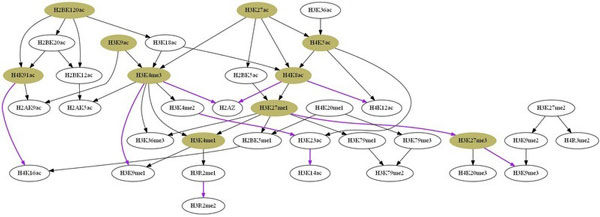
**A network model of highly significant causal relationships given by our method**. 10 most dominant modifications and highest confidence Markov relations are illustrated by filled nodes and purple edges, respectively.

Finally, we assessed whether the proposed method can produce biologically meaningful causal relationships by deriving a network model consisted of the most confident relationships (significance score *≥* 0.7). At this threshold, a network of 49 relationships was created (Figure 6).

We investigated biological characteristics of the resulting network by assessing its *dominant modifications* and the most significant *Markov relations* employing the method described in [20]. By which, dominance score of each modification *X* is calculated by *dScore*(*X*) = Σ*C*_0_(*X*, *Y*)^*k*^, where *C*_0_(*X*, *Y*) denotes the significance score of *X* being an ancestor of *Y*, *k* is the constant to reward highly significant features. Table 4 shows 10 most dominant modifications (*k* = 2, for other values of *k* only the orders were changed) and significant Markov relations, with the corresponding scores given by our method.

**Table 4:** The top dominant histone modifications and significant Markov relations with corresponding dominance and significance scores (***dScore*** and ***C***_**0**_, respectively) given by our method.

Modifications	***dScore***	Markov relations	***C***_0_
*H*3*K*4*Me*3	4.473	*H*3*K*23*Ac → H*3*K*14*Ac*	1
*H*4*K*8*Ac*	2.8836	*H*4*K*8*Ac → H*2*AZ*	1
*H*3*K*27*Me*1	2.7993	*H*4*K*8*Ac → H*4*K*12*Ac*	1
*H*3*K*27*Ac*	2.6593	*H*4*K*91*Ac → H*4*K*16*Ac*	1
*H*4*K*5*Ac*	2.3603	*H*3*K*4*Me*2 *→ H*3*K*23*Ac*	1
*H*2*BK*120*Ac*	2.2473	*H*3*K*4*Me*3 *→ H*2*AZ*	1
*H*4*K*91*Ac*	1.7744	*H*3*K*4*Me*3 *→ H*3*K*9*Me*1	1
*H*3*K*4*Me*1	1.6105	*H*3*R*2*Me*1 *→ H*3*R*2*Me*2	0.99
*H*3*K*27*Me*3	1.5533	*H*3*K*27*Me*3 *→ H*3*K*9*Me*3	0.98
*H*3*K*9*Ac*	1.3325	*H*3*K*27*Me*1 *→ H*3*K*27*Me*3	0.96

Analyzing the top dominant modifications, we found that 8 out of 10 PTMs, {*H*3*K*4*Me*3, *H*3*K*27*Ac*, *H*2*BK*120*Ac*, *H*4*K*8*Ac*, *H*4*K*5*Ac*, *H*4*K*91*Ac*, *H*3*K*4*Me*1, *H*3*K*9*Ac*}, have been reported in the original research as important marks that appeared in the modification back-bone at promoters [8]. For the other two, *H*3*K*27*Me*3 is known as an important repressive mark, and *H*3*K*27*Me*1 as an active mark at promoters [9]. Interestingly, the result suggested the significant role of *H*2*BK*120*Ac* and its regulatory effect on *H*3*K*4*Me*3, an important modification mark of active promoters, through the chain *H*2*BK*120*Ac → H*3*K*18*Ac → H*3*K*4*Me*3. For a long time, the functions of *H*2*B* modifications, particularly *H*2*BK*120*Ac*, have remained obscure compared to other modifications [56]. Just recently there has been an indication that *H*2*BK*120*Ac* appears as an early modification mark in TSS regions and affects *H*2*BK*120*Ub* [57], a modification that regulates *H*3*K*4*Me*3 [58, 59], providing support for our finding. Investigation of the most significant Markov relations revealed that well-characterized modifications are mostly functionally related. For example, the N-terminal tail of histone H4 has four acetylated lysines: K5, K8, K12, K16, of which H4 K5Ac/K8Ac/K12Ac play a non-specific, cumulative regulatory role different from that of H4K16Ac [60]. In consistence with this observation, these modifications were predicted to be closely linked and separated from *H*4*K*16*Ac* in the resulting model: *H*4*K*5*Ac → H*4*K*8*Ac*, *H*4*K*5*Ac → H*4*K*12*Ac*, and *H*4*K*8*Ac → H*4*K*12*Ac* (one of the top 10 Markov relations). For other less well-known modifications, such as *H*3*R*2 methylations or H3K27 mono-methylation, the links might suggest novel biological understanding. While the relationship between *H*3*R*2*Me*1 *→ H*3*R*2*Me*2 might reflect a directional equilibrium between mono- and di-methyl *H*3*R*2, the one between *H*3*K*27*Me*1 *→ H*3*K*27*Me*3 might reflect their functional association through G9a methyltransferase, as recently reported by [61]. More interestingly, 4 out of 10 most significant Markov relations have already been reported to be causal in literature. [14] have shown evidences for causal relationships of *H*3*K*27*Me*3 *→ H*3*K*9*Me*3 and *H*3*K*4*Me*3 *→ H*2*AZ*. In [62], *H*3*K*9*Me*1*/*2 was shown to be demethylated by *P HD* finger protein 8 (*PHF*8), whose catalytic activity is in turn stimulated by *H*3*K*4*Me*3, suggesting the causal effect of *H*3*K*4*Me*3 on *H*3*K*9*Me*1, represented by the link *H*3*K*4*Me*3 *→ H*3*K*9*Me*1. Also, the deposition of histone variant *H*2*A.Z* by *SWR*1 complex is known to be triggered by *NuA*4-mediated acetylation of histone *H*4 [63, 64]. Our model supported this observation with the relationship *H*4*K*8*Ac → H*2*AZ*. Additionally, causal effects have also been observed to support other relationships of the resulting model. For example, [14] have given evidence for the relationship *H*3*K*4*Me*3 *→ H*3*K*36*Me*3. [65] have reported that the recruitment of *MLL*1, a histone methyltransferase responsible for *H*3*K*4 methylation, is required for the binding of *TIP*60 histone acetyltransferase, which catalytically acetylates *H*2*AK*5. In agreement, our model predicted the relationship *H*3*K*4*Me*3 *→ H*2*AK*5*Ac*, suggesting causal effect of *H*3*K*4*Me*3 on *H*2*AK*5*Ac*.

## Conclusion

Elucidation of functional relationships among histone modifications is crucial to understanding important chromatin-mediated processes. Previous BN-based approaches, however, have not taken into account the existence of hidden regulators when inferring causal relationships of PTMs. We tackled the problem by proposing a novel approach that exploits chromatin organizational information to capture the effect of PTM hidden regulators. Application on human epigenomic data showed the advantage of the proposed method over the previous ones. Moreover, it could recover biologically relevant causal relationships between histone modifications, which may be useful for future investigation of histone crosstalk.
